# Biological properties of α-actinin-2 and its role and mechanisms in disease development

**DOI:** 10.3389/fphys.2026.1794324

**Published:** 2026-04-23

**Authors:** Juan Meng, Shu-qiong Xu, Qi Deng, Ling-ling Jiang, Dan-Ping Huang, Qing-song Wang, Yong-Kang Wu

**Affiliations:** 1 Department of Pediatrics, West China Hospital Sichuan University Jintang Hospital, Jintang First People’s Hospital, Chengdu, China; 2 Department of Gastroenterology, West China Hospital Sichuan University Jintang Hospital, Jintang First People’s Hospital, Chengdu, China; 3 Department of Laboratory Medicine, West China Hospital, Sichuan University, Chengdu, China; 4 West China Hospital Sichuan University Jintang Hospital, Jintang First People’s Hospital, Chengdu, China

**Keywords:** ACTN2, biomarker, cardiomyopathy, sarcomere, therapeutic target, α-actinin-2

## Abstract

α-Actinin-2 (encoded by the *ACTN2* gene) is a critical cytoskeletal protein predominantly expressed in skeletal and cardiac muscle, where it anchors actin filaments to the sarcomeric Z-disk. While its structural role is well-established, emerging evidence increasingly implicates *ACTN2* dysfunction in a spectrum of disorders, including cardiomyopathies, distal myopathies, and neurodegenerative conditions. Despite these established associations, a systematic synthesis of the structure-function relationship of α-Actinin-2 and its pathogenic mechanisms remains lacking. This review comprehensively summarizes the multifaceted roles of α-Actinin-2, ranging from its molecular architecture and cytoskeletal regulation to its involvement in diverse signaling pathways. Furthermore, we establish an analytical framework connecting specific genetic variants to clinical phenotypes, thereby providing theoretical insights to facilitate the identification of diagnostic biomarkers and the development of targeted therapeutic strategies.

## 1 Introduction

α-Actinin-2 belongs to the spectrin superfamily of actin-binding proteins (Lek et al., 2010). Originally identified as Z-disk proteins responsible for actin cross-linking in muscle tissue (Ichinoseki-Sekine et al., 2012; Kostan et al., 2021; Sbai et al., 2021), this family comprises four closely related paralogs (α-actinin-1 through -4). While these isoforms share conserved biological functions supporting cellular architecture (Lin et al., 1998; Tousley et al., 2019; Hayes et al., 2025), α-actinin-2, encoded by the *ACTN2* gene, exhibits a unique functional localization. It serves as a primary structural component of the sarcomeric Z-disk, where it maintains sarcomere integrity by specifically cross-linking actin filaments in an antiparallel manner (Sponga et al., 2021; Wadmore et al., 2021; Ranta-Aho et al., 2022). Beyond this canonical structural function, recent studies have uncovered its role as a molecular hub integrating mechanical stress with intracellular signaling networks, such as ion channel gating and transcriptional regulation (Huang et al., 2006; Oikonomou et al., 2011; Gupta et al., 2012).

Despite the identification of numerous pathogenic mutations in the *ACTN2* gene associated with cardiomyopathies and myopathies, a systematic synthesis linking these specific structural alterations to diverse clinical phenotypes is currently lacking. To address this gap, this review comprehensively summarizes the biological properties of α-actinin-2 and establishes an analytical framework connecting genetic variants to disease mechanisms, aiming to facilitate the development of targeted therapeutic strategies (Guy et al., 1999; Mills et al., 2001; Yang et al., 2003).

To provide a comprehensive overview, we conducted a systematic literature search in PubMed databases from 1993 to 2025. Key search terms included “α-actinin-2”, “*ACTN2*”, “cardiomyopathy”, “skeletal muscle”, and “signal transduction”. We prioritized studies focusing on the molecular mechanisms, genetic mutations, and clinical phenotypes associated with α-actinin-2, while excluding articles not published in English or lacking full text. **Supplementary Figure S1** illustrates the publication trend based on these criteria.

## 2 Structural features and functional mechanisms of α-actinin-2

### 2.1 Structural features of α-actinin-2

**Figure 1:**
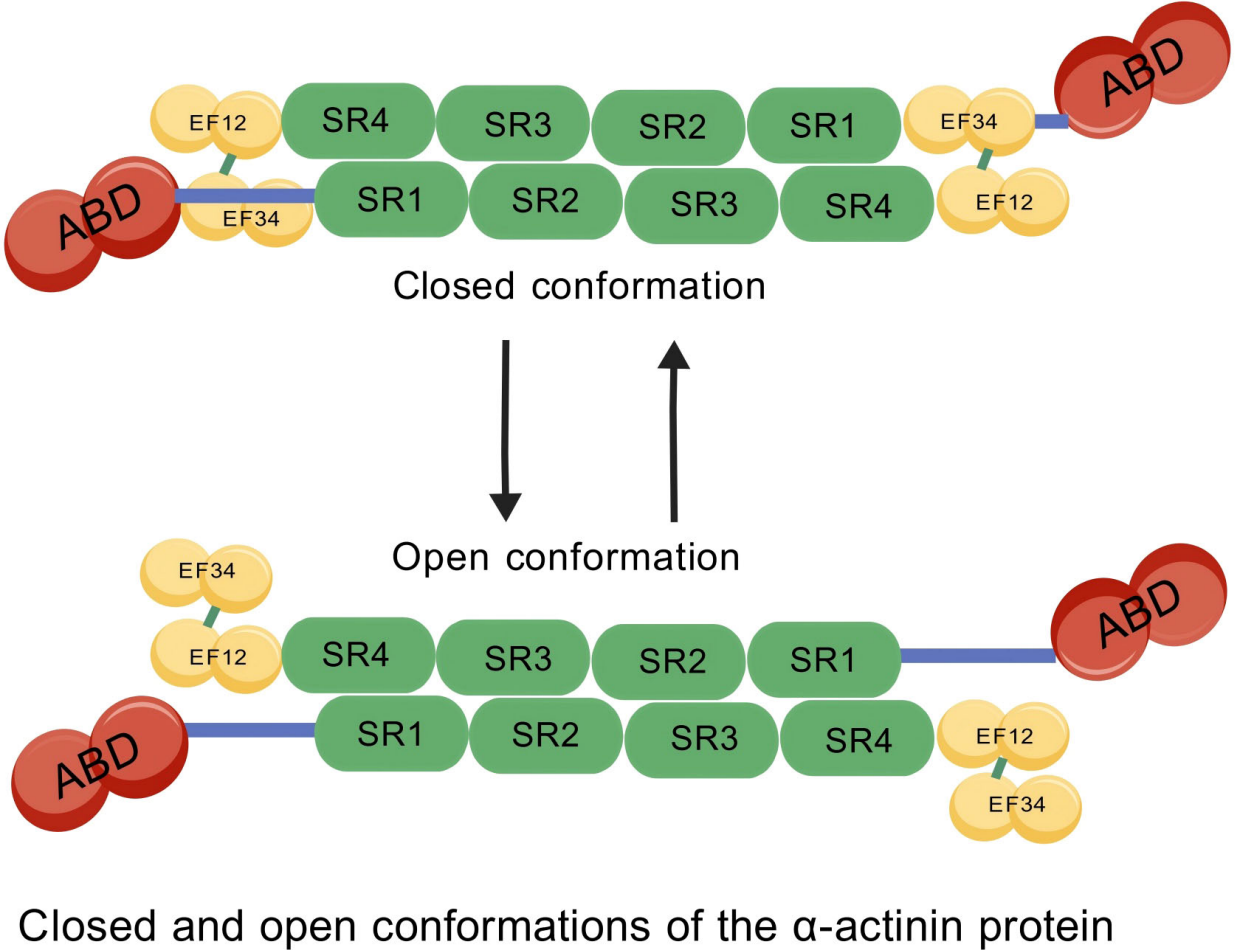
Schematic diagram of the molecular structure and conformational states of α-actinin-2. As reported by Euripedes de Almeida Ribeiro Jr et al. in 2014 (PMID: 25433700), α-actinin-2 exists in both closed and open conformations and functions as a counter-parallel homodimer with a molecular weight exceeding 200 kDa. Structurally, it consists of four distinct segments corresponding to functional domains: the green segment represents the four spectrin-like repeat (SR) sequences in the central region, the red segment denotes the N-terminal actin-binding domain (ABD), the yellow segment corresponds to the two pairs of EF-hand motifs (EF) within the C-terminal calmodulin-like domain (CAMD), and the blue segment is the neck region connecting these functional domains. This structural organization endows α-actinin-2 with the capabilities of binding actin filaments, forming antiparallel homodimers, and regulating calcium levels, which lays the foundation for its physiological functions.

In 2014, Euripedes de Almeida Ribeiro Jr et al. reported that α-actinin-2 functions as an antiparallel homodimer of over 200 kDa (PMID: 25433700), composed of an N-terminal actin-binding domain (ABD), a central region containing four spectrin-like repeat sequences(SR1/SR2/SR3/SR4), and a C-terminal calmodulin-like domain (CAMD) with two pairs of EF-hand motifs (EF)(**Figure 1**). This unique structural organization enables α-actinin-2 to bind actin filaments, form antiparallel homodimers, and regulate calcium levels through its EF-hand domains. The conservation and diversity of these domains provide the foundation for their functional roles across a wide range of cellular environments (Atkinson et al., 2001; Klaavuniemi et al., 2004; Addario et al., 2011; Hsu et al., 2018).

Furthermore, α-actinin-2 exists in a dynamic equilibrium between a “closed” and an “open” conformation. Functionally, the closed state represents an auto-inhibited configuration where the C-terminal CAMD interacts closely with the neck region. This auto-inhibition sterically limits the protein’s ability to bind certain ligands. Conversely, upon specific physiological triggers—such as binding to PIP2 or interactions with mechanical stress-sensing proteins—the molecule shifts to the open state. This conformational transition exposes previously hidden binding interfaces, drastically increasing its affinity for various downstream sarcomeric and signaling partners.

While the individual functions of α-actinin-2 are well-documented, an integrative analysis reveals that its physiological output is strictly dictated by domain-specific availability. The N-terminal ABD is the primary driver of static structural stability in the Z-disk, whereas the C-terminal EF-hands act as dynamic sensors for intracellular signaling (e.g., CaMKII, NMDA receptors). Crucially, recent structural data suggest that these domains are not functionally isolated; mutations in the ABD can allosterically impact the conformation of the central rod domain, thereby altering its binding affinity for signaling partners. This structural crosstalk implies that α-actinin-2 is not merely a passive scaffold but an active mechanotransducer that converts mechanical stress (via the rod domain) into biochemical signals (via the EF-hands).

This illustrates the trend in the number of α-actin-2-related studies within the PubMed database from 1993 to 2025, providing data support for our in-depth analysis of this trend.

### 2.2 Functional mechanism of α-actinin-2

#### 2.2.1 Myosin filament assembly and maintenance

**Figure 2:**
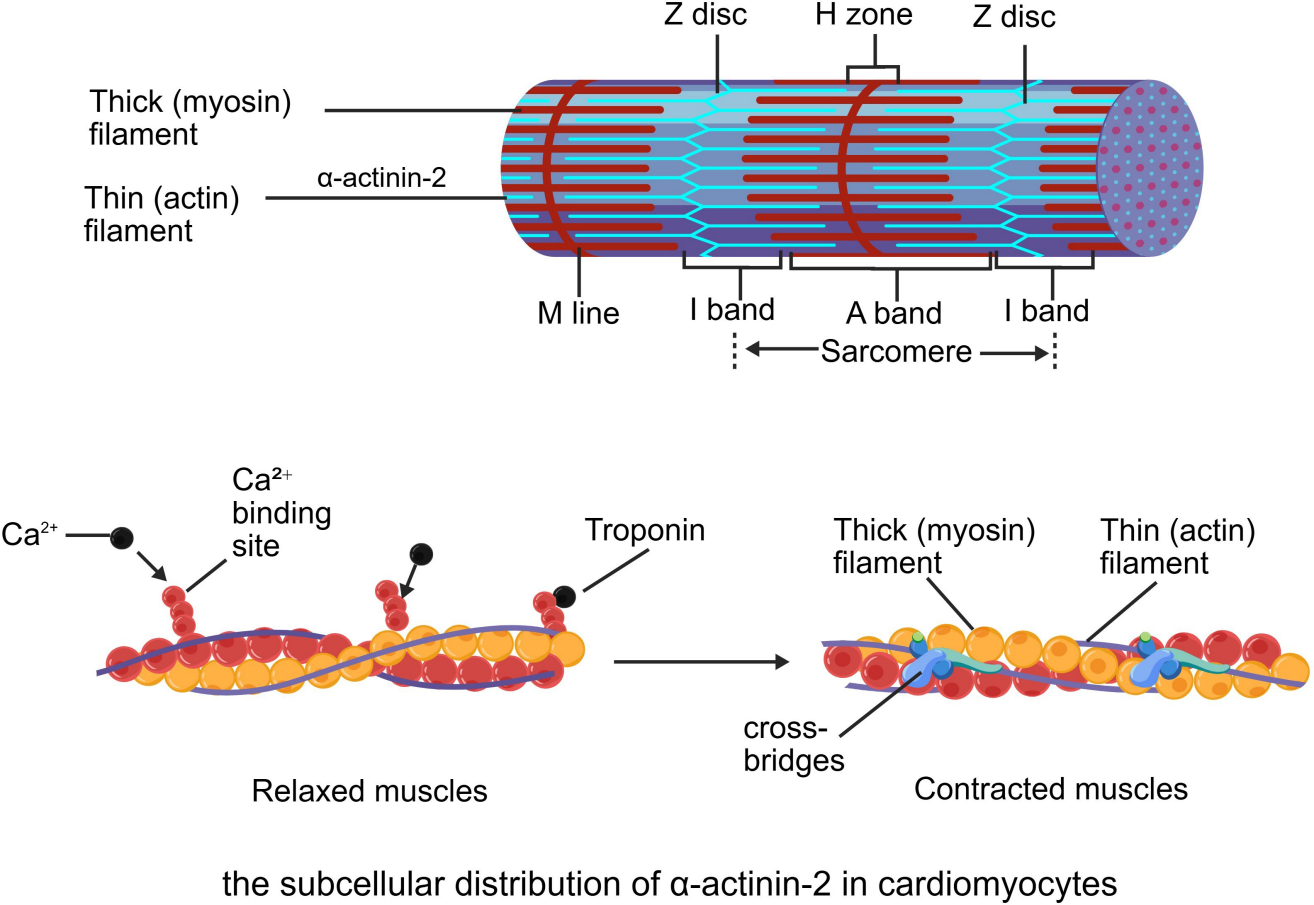
Subcellular distribution of α-actinin-2 in cardiomyocytes and the structural basis of muscle contraction. (Top) Schematic of a cardiac sarcomere illustrating the precise localization of α-actinin-2 specifically at the Z-discs. In this panel, the blue thin lines represent the Z-discs and the anchored thin (actin) filaments, whereas the red thick lines indicate the thick (myosin) filaments within the A-band. α-actinin-2 acts as a crucial cross-linker at the Z-discs to maintain the structural integrity of the sarcomere. (Bottom) Molecular view of the contractile apparatus transitioning from a relaxed state to a contracted state, highlighting the sliding interaction between thick (myosin) and thin (actin) filaments driven by calcium binding and cross-bridge formation.

α-Actinin-2 plays a crucial role in myosin assembly and maintenance. As the fundamental unit of muscle contraction, the structural integrity and function of myosin are essential for normal muscle activity (Maselli et al., 2002; Ponomareva and Ogneva, 2012),. Specifically, α-actinin-2 anchors actin filaments to the Z-disk, thereby preserving the structural integrity of the sarcomere. This anchoring process is vital for the contractile function of muscle cells and for maintaining overall muscle stability (Lu et al., 2022; Neininger-Castro et al., 2023a; Neininger-Castro et al., 2023b). A schematic representation of this subcellular architecture is provided in **Figure 2**, which visually summarizes the specific localization of α-actinin-2 at the Z-disc and the dynamic process of sarcomere contraction.

A crucial function of α-actinin-2 is to anchor actin filaments to the Z-disk. This anchoring not only contributes to the formation of a stable sarcomere structure but also serves as the foundation for the transmission of muscle contraction force (Xu et al., 2024). In this process, α-actinin-2 interacts with actin filaments through its ABD, ensuring proper alignment and anchoring of actin filaments (Chopra et al., 2018; Silviana et al., 2021).

In addition to its role in the initial assembly of sarcomeres, α-actinin-2 participates in sarcomere maturation and remodeling processes (Steiner-Champliaud et al., 2010). Segment maturation involves structural optimization and functional enhancement, whereas remodeling responds to muscle injury or changing physiological demands (Day et al., 2024; Watanabe and Wada, 2025). The role of α-actinin-2 in these processes, which promote segment stability and functionality, is evident under diverse physiological conditions, such as during postnatal cardiac maturation and skeletal muscle adaptation to exercise training (Norman et al., 2009; Zhang et al., 2016; Tyganov et al., 2021; Noureddine et al., 2025).

In cardiomyocytes, α-actinin-2 provides essential structural coupling by directly interacting with dystrophin, a core component of the Dystrophin-Glycoprotein Complex (DGC) (Hance et al., 1999; Xu et al., 2024). The DGC physically connects the intracellular cytoskeleton to the extracellular matrix. During repeated cycles of muscle contraction, this connection provides mechanical stability to the sarcolemma, protecting the cell membrane from contraction-induced damage (Harper et al., 2002).By binding to dystrophin, α-actinin-2 anchors the DGC at the costameres along the Z-disk. This protein-protein interaction is required for the spatial invagination and structural maintenance of T-tubules. Consequently, anchoring the T-tubule membrane via dystrophin ensures the proper alignment of T-tubules with the sarcoplasmic reticulum, establishing the structural basis for excitation-contraction coupling in the heart (Pecorari et al., 2020; Tsai et al., 2021).

#### 2.2.2 Signal transduction

**Figure 3:**
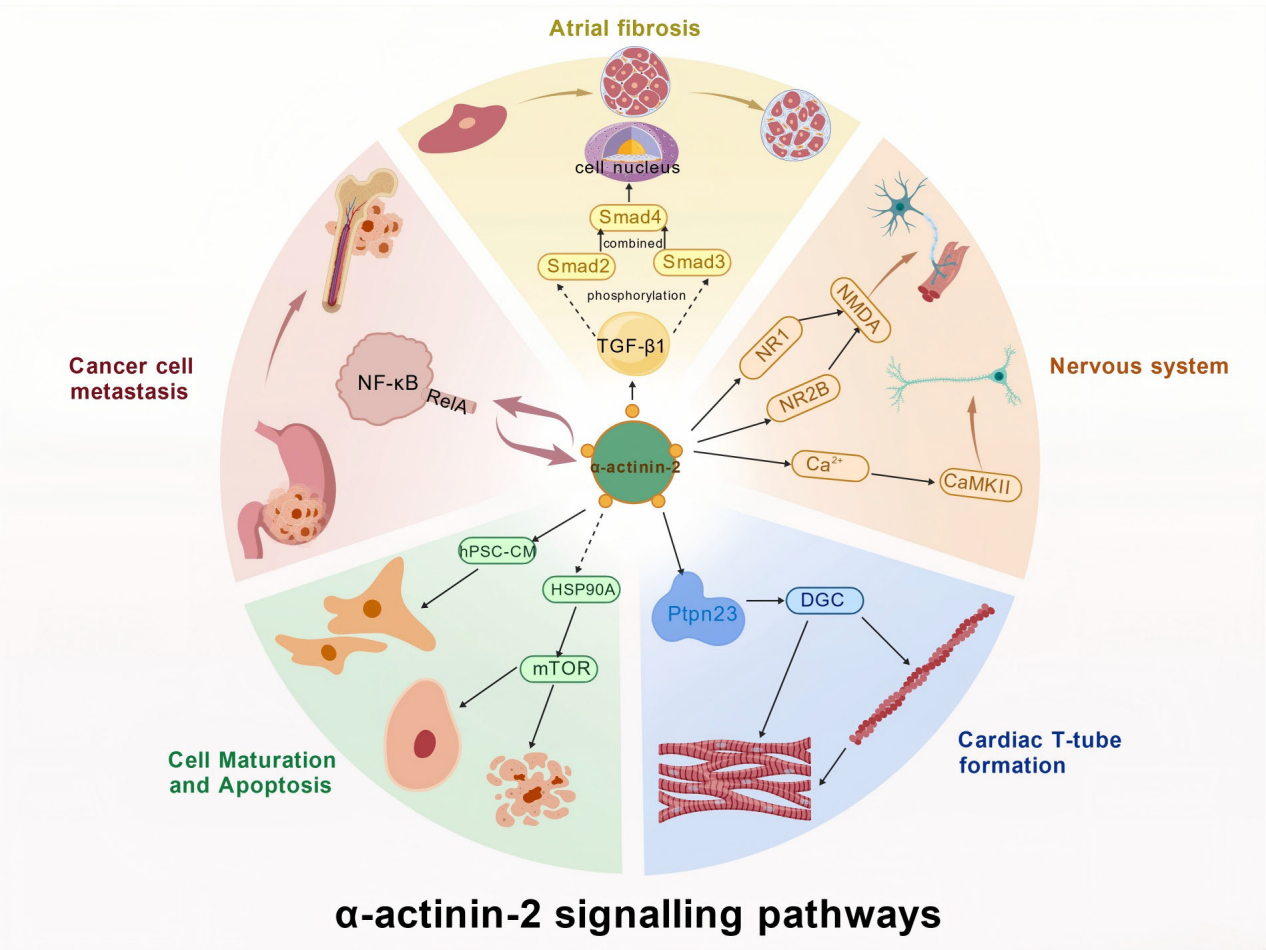
α-actinin-2 functions as a pleiotropic signaling scaffold integrating diverse physiological pathways. The schematic illustrates how α-actinin-2 coordinates distinct downstream effects across different tissues: (Top, Atrial Fibrosis) it exacerbates fibrosis by enhancing TGF-β1/Smad signaling; (Right, Nervous System) it regulates synaptic plasticity via interactions with NMDA receptors and CaMKII; (Bottom Right, Cardiac Structure) it anchors the Dystrophin-Glycoprotein Complex (DGC) via Ptpn23 to maintain T-tubule architecture; (Bottom Left, Maturation) it promotes cardiomyocyte maturation through the HSP90A/mTOR axis; and (Left, Metastasis) it drives cancer cell migration via an NF-κB positive feedback loop.

α-Actinin-2 not only plays a key role in muscle structure but also participates in various signal transduction processes essential for cellular function and adaptability (Hodges et al., 2014; Chopra et al., 2018; Tyganov et al., 2021; Curtis et al., 2023; Wang et al., 2023; Htet et al., 2024). (**Figure 3**, **Table 1**).

**Table 1:** Mechanisms of alpha-actinin-2 in signal transduction.

Biological process	Signaling pathway/Interaction	Mechanism of action	Experimental model	Ref.
Neuronal Plasticity	NMDA Receptor (NR1, NR2B)	Modulates receptor localization; Regulates synaptic plasticity and signaling efficiency via EF-hand domain binding.	Rat (Hippocampus); Cell culture	(Htet et al., 2024)
Synaptic Potentiation	CaMKII	Activates CaMKII via C-terminal CaM-like domain; Facilitates LTP (Long-term Potentiation) in excitatory synapses.	Rat; Cell culture	(Wang et al., 2023)
T-Tubule Shaping	*Ptpn23*/DGC Complex	Coordinates Dystrophin-Glycoprotein Complex (DGC) assembly at costameres; Maintains T-tubule spatial patterning.	Rat; Cell culture	(Chopra et al., 2018)
Cardiomyocyte Maturation	HSP90A/mTOR Pathway	Promotes maturation and prevents aging via HSP90A induction and mTOR activation.	Rat; iPSC-CMs	(Curtis et al., 2023)
Cancer Metastasis	NF-kappaB (RelA)	Forms positive feedback loop with RelA; Promotes filopodia formation and bone marrow metastasis (Gastric Cancer).	Rat; Cell culture	(Hodges et al., 2014)
Atrial Fibrosis	TGF-β1/Smad	Upregulates TGF-β1/Smad pathway in fibroblasts; Promotes collagen deposition and fibrotic remodeling.	Rat (Atrial fibroblasts)	(Tyganov et al., 2021)

##### 2.2.2.1 Role in cardiomyocytes

Chen Xu et al. investigated the unclear coordination mechanism between DGC assembly and T-tubule formation/maintenance in cardiomyocytes, as well as the unidentified molecular causes of T-tubule abnormalities in dilated cardiomyopathy. Their study incorporated clinical sample analysis (myocardial tissue from patients with dilated cardiomyopathy), gene-edited rat models (cardiac cell-specific *Ptpn23*/*ACTN2* knockout, DmdE4 knockout), AAV9-mediated vacuolar cell mutagenesis, T-tubule structural analysis (staining/EM), and biochemical assays (glycerol gradient centrifugation). They demonstrated that *Ptpn23* maintains costamer integrity by interacting with actin (primarily α-actinin-2) and dystrophin. α-Actinin-2 deficiency changes *Ptpn23*/DGC localization, Dmd deficiency causes T-tubule-like defects, and adult *Ptpn23* knockout also impairs T-tubule maintenance (Xu et al., 2024). These findings establish *Ptpn23* as essential for cardiac T-tubule formation and preservation along the Z-disc. During postnatal cardiac development, *Ptpn23* interacts with myosin alpha-actinin and components of the DGC at the costamere, coordinating their assembly to shape the spatial pattern and morphology of T-tubules.

##### 2.2.2.2 Role in cellular stress responses

Moreover, α-actinin-2 participates in cellular stress responses by interacting with heat shock proteins, thereby protecting cells from oxidative stress-induced damage (Htet et al., 2024). MyoHtet et al. investigated unresolved questions regarding the regulatory mechanisms of cardiomyocyte (CM) maturation, the function of the *ACTN2* enhancer, and the role of cis-regulatory elements in cardiomyopathy. Employing hPSC models, rat models, transcriptomic analysis, and enCRISPRa technology, they identified a core finding: *ACTN2* levels increase with CM maturation. Heterozygous *ACTN2* enhancer deletion causes CM morphological/functional abnormalities and impaired mitochondrial respiration. It also suppresses maturation and promotes aging by inducing heat shock protein 90A, which in turn activates mTOR signaling. This mechanism aids cellular recovery and tissue repair (Domazetovska et al., 2007), especially crucial when muscle or nerve cells are damaged (Day et al., 2024).

##### 2.2.2.3 Exacerbation of atrial cell fibrosis

Zhang Lei et al. investigated the interaction between α-actinin-2 and the TGF-β1/Smad signaling pathway in atrial fibrosis and its effect on atrial fibrillation (AF). The findings demonstrated that, compared with the sinus rhythm group, patients with congenital heart disease and rheumatic heart disease (RHD) exhibited significantly elevated collagen content, increased mRNA and protein expression of α-actinin-2, and increased expression of components of the TGF-β1/Smad signaling pathway. Furthermore, in patients with RHD, mRNA and protein levels of α-actinin-2 and TGF-β1 were positively associated with collagen volume fraction. This positive correlation suggests that α-actinin-2 promotes atrial fibrosis development by upregulating the TGF-β1/Smad signaling pathway in atrial fibroblasts (Zhang et al., 2016).

##### 2.2.2.4 Interaction with signaling molecules

Dunah et al. investigated the localization of α-actinin-2 in the rat striatum. By employing α-actinin-2-specific antibody immunodetection, double-label *in situ* hybridization, and immunoprecipitation of membrane protein extracts, they confirmed the presence of α-actinin-2 within NMDA receptor subunit (NR1, NR2A, and NR2B) hetero-complexes, demonstrating its capacity to interact with NMDA receptors (Dunah et al., 2000). Furthermore, Robison et al. investigated the synaptic targeting mechanism of dendritic CaMKII. Using glutathione-agarose co-precipitation assays, protein binding competition experiments, and multiprotein co-immunoprecipitation validation, they identified residues 819–894 on the C-terminus of α-actinin-2 as the minimal CaMKII-binding domain and demonstrated that α-actinin-2 binds to all CaMKII subtypes (Dunah et al., 2000). These molecules play crucial roles in synaptic plasticity and neurotransmitter release within neurons. Through these interactions, α-actinin-2 modulates neuronal excitability and signaling efficiency, thereby influencing cognitive functions, including learning and memory (Leonard et al., 2002; Robison et al., 2005; Hodges et al., 2014; Curtis et al., 2023).

##### 2.2.2.5 Promotion of gastric cancer bone marrow metastasis

GC complicated by BMM often results in severe hematologic abnormalities, including disseminated intravascular coagulation and microangiopathic hemolytic anemia, representing a highly aggressive subtype of GC. Caiqin Wang et al. investigated the molecular mechanisms underlying α-actinin-2-mediated BMM using multiplex immunofluorescence staining, Western blotting, chromatin immunoprecipitation, and luciferase reporter assays. Their findings revealed that α-actinin-2 binds to the RelA subunit of NF-κB to form heterotrimers, creating a positive feedback regulatory loop that facilitates GC cell metastasis to the bone marrow (Wang et al., 2023).

#### 2.2.3 Cytoskeletal remodeling

α-Actinin-2 is a crucial cytoskeletal protein playing key roles in muscle cells and neurons. It not only participates in dynamic cytoskeletal remodeling, influencing cell morphology and motility (Melnikov et al., 2022), but also exerts central regulatory functions in cell migration, tissue repair, and tumor cell invasion (Sbai et al., 2021). Particularly during tumor metastasis, α-actinin-2 may promote tumor cell migration and invasion by reorganizing the cytoskeleton, underscoring its potential significance in tumor progression (Wang et al., 2023). These functions demonstrate the multifaceted role of α-actinin-2 in maintaining cellular structural integrity and regulating cellular behavior, as well as its potential as a therapeutic intervention target.

##### 2.2.3.1 Actin filament assembly and disassembly

α-Actinin-2 influences cytoskeletal organization and function by regulating actin filament assembly and disassembly, a process critical for muscle contraction and cell migration (Domazetovska et al., 2007). By stabilizing actin filament bundles, α-actinin-2 promotes cytoskeletal integrity and functionality (Lin et al., 2010).

##### 2.2.3.2 Muscle strength and endurance

In skeletal muscle, α-actinin-2 expression levels are strongly associated with muscle strength and endurance (Yang et al., 2003). Furthermore, genetic polymorphisms in this protein may influence athletic performance, further highlighting the critical role of α-actinin-2 in muscular function (Seto et al., 2011; Seto et al., 2013). Notably, variations in α-actinin-2 expression appear to correlate with muscle adaptation to exercise training, emphasizing its crucial role in regulating muscle performance (Ogura et al., 2011).

##### 2.2.3.3 Dendritic spine formation in neurons

In neurons, α-actinin-2 contributes to dendritic spine formation and the assembly of the postsynaptic density (Shirao and Sekino, 2001). Dendritic spines are essential structures for receiving synaptic input and processing information, and changes in their morphology and function are closely linked to learning and memory (Cattabeni et al., 1999; Hodges et al., 2014). By influencing spine structure and stability, α-actinin-2 plays an essential role in neuronal signaling and plasticity (Wyszynski et al., 1997).

##### 2.2.3.4 Cell migration capacity

Besides, α-actinin-2 contributes to regulating cell migration, a process crucial for tissue organization, wound healing, and tumor metastasis (Cooper et al., 2004). By modulating the dynamic reorganization of the cytoskeleton, α-actinin-2 can either promote or inhibit cell movement depending on specific physiological or pathological contexts (Sbai et al., 2021).

##### 2.2.3.5 Changes in cell morphology

Additionally, skeletal remodeling involves alterations in cell morphology, which significantly impact cellular function and behavior (Zhou et al., 2024). By regulating actin filament organization, α-actinin-2 influences cell shape and polarity, thereby affecting directed migration and tissue localization (Guo et al., 2021).

## 3 α-actinin-2 and disease association

Although clinical manifestations of *ACTN2*-related disorders are diverse, encompassing cardiac, skeletal, and neurological systems, they can be mechanistically categorized into two distinct clusters: (1) Mutation-Driven Structuralopathies, where missense or frameshift variants (e.g., in HCM and distal myopathy) directly compromise Z-disk integrity or cause protein aggregation; and (2) Expression-Driven Channelopathies/Signaling Disorders, where aberrant protein levels (e.g., in atrial fibrillation or cancer metastasis) disrupt downstream signaling pathways without altering the protein sequence. The following sections detail these disease associations, structured by clinical phenotype to facilitate translational reference, while highlighting these underlying mechanistic dichotomies.

**Figure 4:**
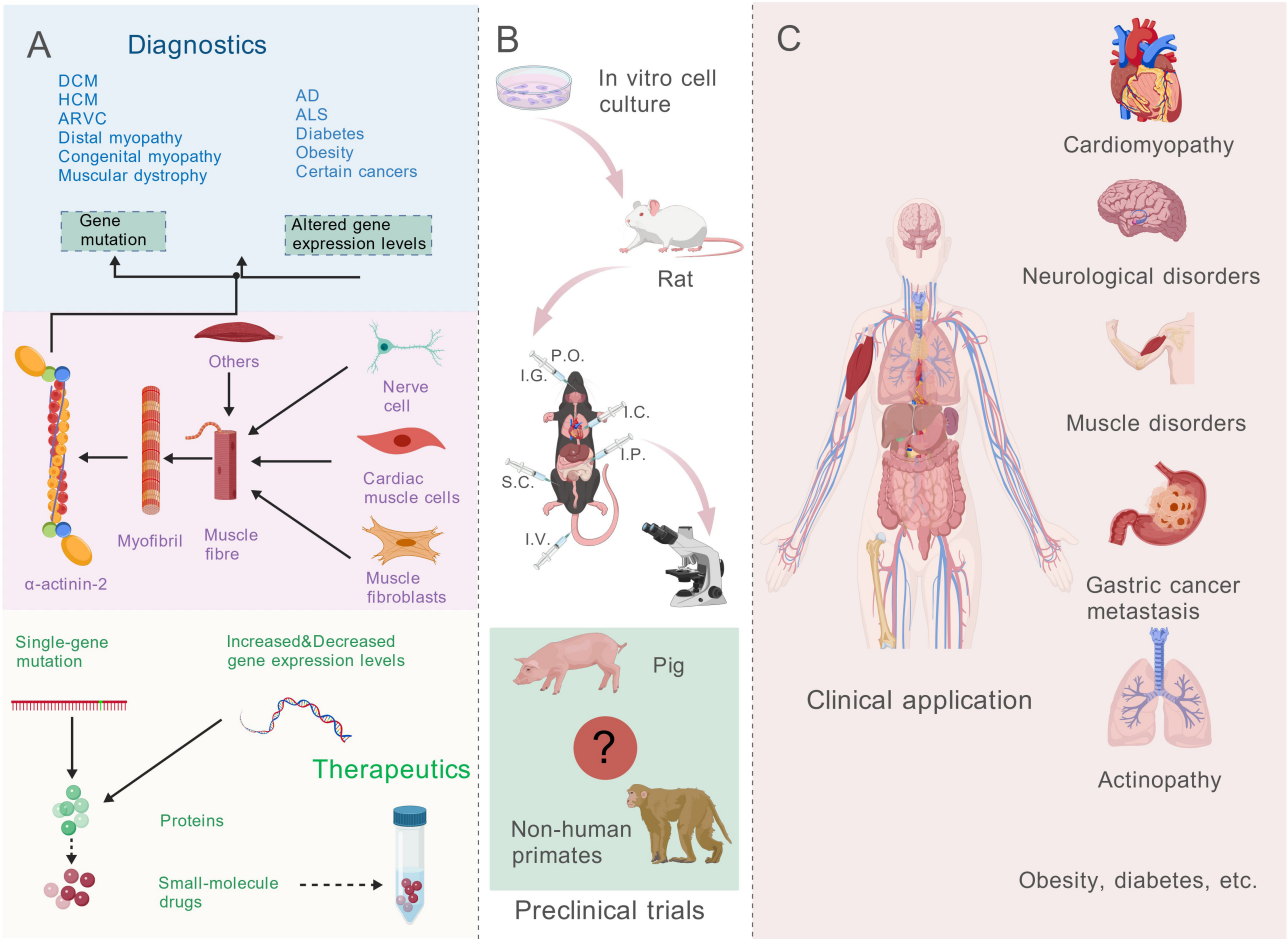
Multidimensional landscape of α-actinin-2 in disease mechanisms, research models, and clinical manifestations. **(A)** Pathogenic Divergence: Distinguishes between structural diseases (cardiomyopathies, myopathies) primarily caused by specific gene mutations (left), and systemic disorders (neurodegenerative, metabolic, cancer) driven by altered expression levels (right). **(B)** Research Model Gaps: Highlights the current reliance on rodent and cell models, pointing out the critical lack of non-human primate models (indicated by “?”) which limits translational precision. **(C)** Systemic Impact: Visualizes the broad clinical spectrum of α-actinin-2 dysfunction, emphasizing its potential as a diagnostic biomarker and therapeutic target across cardiac, neurological, muscular, and metabolic systems.

α-actinin-2 participates in the development and progression of multiple diseases, with its gene mutations or abnormal expression closely associated with the onset of various disorders (**Figure 4A**). Existing studies using mouse cell culture experiments have preliminarily revealed α-actinin-2-related disease mechanisms. However, these investigations have significant limitations and lack human clinical validation (**Figure 4B**).

Furthermore, the expression and distribution patterns of α-actinin-2 across various human organ systems have been experimentally characterized (**Figure 4C**).

Genetic mutations or abnormal expression of α-actinin-2 have been indicated to be closely correlated with the onset and progression of multisystem disorders, including cardiomyopathies, myopathies, neurodegenerative disorders, and certain malignancies. Critically, the strength of evidence linking α-actinin-2 to these disorders varies by disease category. The association between *ACTN2* mutations and striated muscle diseases (cardiomyopathies, distal myopathies) is supported by robust genetic segregation and functional validation, establishing a direct causal link. In contrast, the involvement of α-actinin-2 in neurodegenerative and metabolic disorders is primarily evidenced by altered expression levels (transcriptomic or proteomic data). This suggests a correlative or secondary role, where the protein likely acts as a downstream effector or susceptibility factor rather than a primary genetic driver (Garcia et al., 2023)(**Table 2**).

**Table 2:** Correlation of α-actinin-2 with different diseases.

Disease category	Disease name	Altered gene expression levels	Gene mutations	Pathological features	Related research	References
Cardiomyopathy	Hypertrophic cardiomyopathy (HCM)	Not reported	*p.Met228Thr*, *p.Thr247Met*, *p.Gly111Val*, *p.Ala119Thr*	Myocardial hypertrophy, left ventricular outflow tract obstruction, atrial fibrillation	Research indicates these mutations cause abnormal sarcomere structure, impairing myocardial cell contractility and electrophysiological properties.	(Atang et al., 2023; Broadway-Stringer et al., 2023; Noureddine et al., 2025)
	Dilated cardiomyopathy (DCM)	Not reported	*p.Glu583Ala*, *p.Glu628Gly*	Myocardial dilation, impaired contractility	Research indicates these mutations impair mitochondrial function and energy metabolism, further exacerbating cardiomyocyte damage.	(Chiu et al., 2010; Curd et al., 2021)
	Arrhythmogenic right ventricular cardiomyopathy (ARVC)	Not reported	*p.Arg506Gly*	Right ventricular fibrofatty replacement, arrhythmia	Studies indicate that these mutations impair calcium-binding capacity, leading to abnormal calcium handling in cardiomyocytes and arrhythmias.	(Wechsler and Teichberg, 1998; Good et al., 2020)
	Cardiac Hypertrophy (CH)	Enhanced	Not reported	Myocyte hypertrophy	Research indicates that enhanced α-actinin-2 expression disrupts sarcomere structure and functional homeostasis in cardiomyocytes.	(Li et al., 2004; Xie et al., 2024)
	Arrhythmogenic cardiomyopathy (ACM)	Not reported	*p.Tyr473Cys*	Fibrofatty replacement of cardiomyocytes and altered localization of cardiac ion channels	Research indicates these mutations cause abnormal sarcomere structure, impairing myocardial cell contractility and electrophysiological properties.	(Chen et al., 2019)
	Restrictive cardiomyopathy (RCM)	Not reported	p.Q830Hfs*73	The ventricular wall becomes thickened and hardened, with extremely poor compliance.	Research indicates these mutations lead to abnormal ACTN2 protein accumulation and disrupt sarcomere relaxation, resulting in markedly increased myocardial stiffness and severe diastolic dysfunction.	(Wang et al., 2025)
Myopathy	Distal Myopathy	Not reported	p.Phe835Serfs*66	Distal muscle weakness and atrophy	Research indicates that these mutations cause truncation or loss of function of the α-actinin-2 protein, affecting sarcomere stability and muscle contraction.	(Ranta-Aho et al., 2024a; Ranta-Aho et al., 2024a)
	Congenital myopathy	Not reported	*p.Ile134Asn*	Muscle weakness, reduced muscle tone	Research indicates these mutations impair α-actinin-2’s binding capacity to actin filaments, leading to abnormal sarcomere structure and muscle weakness.	(Lornage et al., 2019; Iruzubieta et al., 2025)
	Muscular dystrophy	Not reported	*p.Cys487Arg*	Reduced sarcolemmal stability, muscle damage	Studies indicate these mutations impair α-actinin-2’s calcium ion binding capacity, leading to decreased sarcolemmal stability and muscle damage.	(Iruzubieta et al., 2025)
Neurodegenerative Diseases	Alzheimer’s disease (AD)	Reduced expression levels	Not reported	Synaptic dysfunction, cognitive impairment	Research indicates that decreased α-actinin-2 expression leads to synaptic dysfunction and cognitive deficits.	(Ratzliff and Soltesz, 2001; Rycroft and Gibb, 2002; Palop et al., 2005)
	Amyotrophic lateral sclerosis (ALS)	Abnormal expression levels	Not reported	Degenerative changes at the neuromuscular junction	Research indicates that abnormal expression levels of α-actinin-2 lead to degenerative changes at the neuromuscular junction.	(Chen et al., 2024)
Other	Diabetes	Decreased expression levels	Not reported	Insulin resistance, reduced glucose uptake	Research indicates that decreased α-actinin-2 expression affects insulin signaling pathways, leading to insulin resistance.	(Hwang et al., 2010; Coletta and Mandarino, 2011)

**Figure 5:**
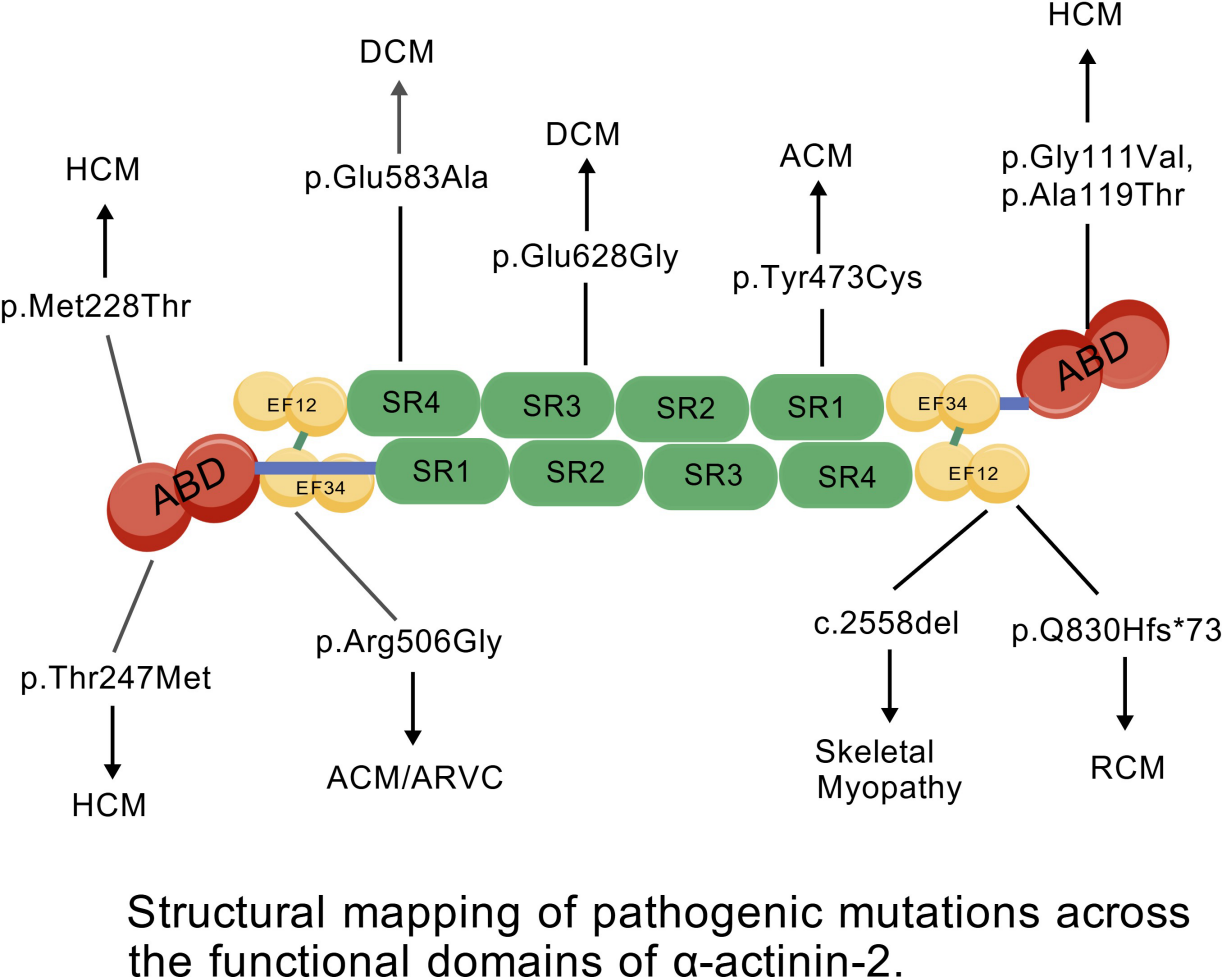
Structural mapping of pathogenic mutations across the functional domains of α-actinin-2. The schematic illustrates the distinct spatial distribution of disease-associated mutations. Structural perturbations within the actin-binding domain (ABD; *p.Met228Thr*, *p.Thr247Met*, *p.Gly111Val*, *p.Ala119Thr*) are predominantly linked to hypertrophic cardiomyopathy (HCM). Meanwhile, variants in the central rod domain (SR1–SR4; *p.Glu583Ala*, *p.Glu628Gly*, *p.Tyr473Cys*) correlate with dilated (DCM) and arrhythmogenic (ACM) cardiomyopathies. Mutations that alter the EF-hand motifs (*p.Arg506Gly*) or cause extreme C-terminal truncations (p.Q830Hfs*73, *c.2558del*) strongly drive arrhythmogenic right ventricular cardiomyopathy (ARVC), severe restrictive cardiomyopathy (RCM), and skeletal myopathies.

To provide a clear genotype-phenotype visual summary, the specific spatial distribution of these identified pathogenic mutations across the functional domains of the α-actinin-2 dimer is illustrated in **Figure 5**.

### 3.1 Cardiomyopathy

Mutations in the α-actinin-2 gene are correlated with various cardiomyopathies, including hypertrophic cardiomyopathy (HCM), dilated cardiomyopathy (DCM), and arrhythmogenic right ventricular cardiomyopathy (ARVC) (Bauce et al., 2000). These mutations typically lead to abnormal sarcomere structure, impairing cardiac muscle cell contraction, function, and electrophysiological properties (Oikonomou et al., 2011; Bagnall et al., 2014; Zech et al., 2022).

#### 3.1.1 Hypertrophic cardiomyopathy

HCM is a hereditary heart disease characterized by myocardial hypertrophy, with its pathogenesis often associated with mutations in sarcomeric protein genes (Cirino et al., 1993-2026). Among these, missense mutations in the α-actinin-2 gene, including *p.Met228Thr* (Noureddine et al., 2025) and *p.Thr247Met* (Atang et al., 2023), have been repeatedly reported to be closely associated with HCM development. Furthermore, recent comprehensive reviews have expanded this mutational landscape, identifying additional variants such as *p.Gly111Val* and *p.Ala119Thr* that are also robustly linked to HCM (Broadway-Stringer et al., 2023).These mutations primarily occur within the ABD or spectrin-like repeat sequences, leading to defects in sarcomere assembly and cardiomyocyte hypertrophy (Lindholm et al., 2021). Specifically, the *p.Met228Thr* mutation within the ABD significantly impairs α-actinin-2’s binding capacity to actin filaments (Girolami et al., 2014). This altered binding not only disrupts normal sarcomere structure but also abnormally improves cardiomyocyte contractility, playing a crucial role in HCM pathogenesis (Haywood et al., 2016; Prondzynski et al., 2019; Broadway-Stringer et al., 2023).

#### 3.1.2 Dilated cardiomyopathy

Dilated cardiomyopathy is a cardiac disease characterized by myocardial dilation and impaired contractility, often resulting in heart failure (Fan et al., 2019; Yang et al., 2019). Mutations in the α-actinin-2 gene, including *p.Glu583Ala* (Curd et al., 2021) and *p.Glu628Gly* (Chiu et al., 2010), have been linked with DCM. These mutations typically affect the rod-like domain of α-actinin-2, leading to instability of the myosin filament and reduced contractility in cardiomyocytes. For instance, the *p.Glu583Ala* mutation resides within the rod-like domain, impairing α-actinin-2 dimerization and consequently affecting its localization and function within myosin filaments (Curd et al., 2021). Further studies indicate that these mutations may exacerbate cardiomyocyte damage by impairing mitochondrial function and energy metabolism (Hershberger et al., 1993-2026; Au et al., 2004; Fan et al., 2018; Guo et al., 2021; Htet et al., 2024).

#### 3.1.3 Arrhythmogenic cardiomyopathy/arrhythmogenic right ventricular cardiomyopathy

ARVC is a cardiac disease characterized by right ventricular fibro-fatty replacement and arrhythmias, often leading to sudden cardiac death (Bauce et al., 2000). Mutations in the α-actinin-2 gene, such as *p.Arg506Gly*, have been linked with ARVC. These mutations typically affect the EF-hand domain of α-actinin-2, changing its sensitivity to calcium ions and consequently affecting the electrophysiological properties of cardiomyocytes (Donkervoort et al., 2024). The *p.Arg506Gly* mutation resides within the EF-hand domain, impairing α-actinin-2’s calcium-binding capacity and leading to abnormal calcium handling and arrhythmias in cardiomyocytes (Wechsler and Teichberg, 1998; Good et al., 2020). Recent clinical evidence has expanded this spectrum; specific variants such as *p.Tyr473Cys* have been associated with left-dominant ACM, where altered localization of cardiac ion channels drives severe electrical instability and early-onset arrhythmias (Chen et al., 2019).

#### 3.1.4 Atrial fibrillation

AF is the most common chronic arrhythmia. AF, a hallmark of atrial structural remodeling, plays a key role in AF, presenting a significant disorder burden on patients (Ahmad et al., 2023). Recent studies suggest that α-actinin-2 variants may also be correlated with malignant arrhythmias in the absence of overt structural heart disorder (Hou and Cortez, 2022). Studies have indicated that α-actinin-2 is upregulated in atrial fibroblasts through the transforming growth factor-β1 (TGF-β1)/Smad pathway, suggesting its potential involvement in TGF-β1/Smad-mediated atrial fibrosis in patients with AF (Girolami et al., 2014; Zhang et al., 2016; Prondzynski et al., 2019; Dorian et al., 2024).

#### 3.1.5 Cardiac hypertrophy

CH serves as an early clinical marker of heart failure progression. In hypertrophic hearts, *ACTN2* (alpha-actinin-2) contributes to disease development through the “circ_Larp4b/miR-298-5p/Mef2c” regulatory axis. In a CH model established by treating HL-1 cells with angiotensin II (Ang II), circ_Larp4b expression was significantly upregulated. By targeting and inhibiting miR-298-5p, it relieved miR-298-5p’s suppression of its downstream target gene Mef2c (myofibrillar muscle enhancer 2), resulting in elevated Mef2c expression. Elevated Mef2c further disrupts *ACTN2* protein expression, alters sarcomere organization, and disturbs cardiomyocyte function, ultimately promoting hypertrophic phenotypes (cell enlargement, upregulation of CH hallmark molecules such as β-MHC/ANP). Conversely, downregulating circ_Larp4b reverses this regulatory cascade and suppresses CH progression. These findings demonstrate that *ACTN2* functions as a key downstream effector mediating CH pathology through this pathway (Li et al., 2004; Xie et al., 2024).

#### 3.1.6 Restrictive cardiomyopathy

Restrictive cardiomyopathy (RCM) is a rare but severe cardiac disorder characterized by markedly increased myocardial stiffness and profound diastolic dysfunction, typically with preserved early systolic capacity. Recent clinical reports have expanded the phenotypic spectrum of ACTN2 mutations to include RCM. Novel frameshift mutations, such as p.Q830Hfs*73, severely compromise Z-disk integrity and cause toxic protein aggregation. Research indicates these mutations lead to abnormal ACTN2 protein accumulation and disrupt sarcomere relaxation, fundamentally impairing ventricular compliance and driving the progressive diastolic heart failure characteristic of RCM (Wang et al., 2025).

### 3.2 Myopathies

α-Actinin-2 gene mutations are also associated with various skeletal muscle diseases, including distal myopathies, congenital myopathies, and muscular dystrophies. These conditions typically manifest as muscle weakness, atrophy, and movement disorders (Harazi et al., 2017; Day et al., 2024; Iruzubieta et al., 2025).

#### 3.2.1 Distal myopathies

Distal myopathies are a group of hereditary muscle diseases characterized by weakness and atrophy of distal limb muscles, with onset associated with diverse genetic variations. The reported *p.Phe835Serfs66* mutation (Ranta-Aho et al., 2024a) is a frameshift mutation that truncates the α-actinin-2 protein. Such mutations typically lead to loss of α-actinin-2 function. Its normal localization at the sarcomere Z-disk is disrupted, affecting its ability to cross-link effectively and anchor actin filaments. This destabilizes the sarcomere structure and interferes with muscle contraction (Chen et al., 2021), ultimately resulting in characteristic clinical phenotypes, including abnormal Z-line structure in muscle fibers and distal muscle weakness (Ranta-Aho et al., 2022).This preferential involvement of distal muscles may be attributed to the higher mechanical stress and eccentric loads placed on these muscles during locomotion, which renders them more susceptible to Z-disk instability caused by α-actinin-2 dysfunction.

#### 3.2.2 Congenital myopathies

Congenital myopathies are a group of hereditary muscle diseases manifesting as muscle weakness and decreased muscle tone at birth or during infancy. Mutations in the α-actinin-2 gene, including *p.Ile134Asn*, have been correlated with congenital myopathies (Iruzubieta et al., 2025). These mutations typically impact the ABD or (rod-like) domain of α-actinin-2, resulting in defects in sarcomere assembly and myofibrillar dysplasia. For instance, the *p.Ile134Asn* mutation resides within the N-terminal ABD, impairing α-actinin-2’s ability to bind actin filaments and leading to abnormal sarcomere structure and muscle weakness (Lornage et al., 2019).

#### 3.2.3 Muscular dystrophy

Dystrophy is a group of hereditary muscle disorders characterized by decreased sarcolemmal stability and muscle damage (Ohsawa et al., 2011). Mutations in the α-actinin-2 gene, including *p.Cys487Arg*, have been linked to dystrophy (Iruzubieta et al., 2025). These mutations typically affect the EF-hand domain of α-actinin-2, changing its sensitivity to calcium ions and, consequently, impacting sarcolemmal stability and muscle contraction function (Vainzof et al., 2002). For instance, the *p.Cys487Arg* mutation resides within the EF-hand domain, impairing α-actinin-2’s calcium-binding capacity and resulting in reduced sarcolemmal stability and muscle damage (Bouju et al., 1999; Faulkner et al., 2001; Hiroi et al., 2008).

### 3.3 Neurodegenerative diseases

Besides, the role of α-actinin-2 in neurodegenerative disorders has drawn attention. In Alzheimer’s disease (AD) models, decreased α-actinin-2 expression associates with synaptic dysfunction and cognitive deficits (Oikonomou et al., 2011). Additionally, α-actinin-2 is implicated in neuromuscular junction degeneration in amyotrophic lateral sclerosis (ALS), where its abnormal expression at the neuromuscular junction may contribute to motor neuron degeneration and muscle atrophy (Sobierajski et al., 2025).

#### 3.3.1 AD

AD is a neurodegenerative disorder characterized by memory loss and cognitive decline. Studies indicate reduced expression of α-actinin-2 in AD models, resulting in synaptic dysfunction and cognitive deficits (Ratzliff and Soltesz, 2001; Rycroft and Gibb, 2002). α-Actinin-2 modulates NMDA receptor localization and activity by binding to its NR1 and NR2B subunits through its EF-hand domain (Rao et al., 1998; Wyszynski et al., 1998). In AD models, decreased α-actinin-2 expression leads to abnormal NMDA receptor localization, impairing synaptic plasticity and signaling in neurons (Allison et al., 1998; Luthi-Carter et al., 2003; Palop et al., 2005; Shin et al., 2005; Bouhamdan et al., 2006; Wang et al., 2008).

#### 3.3.2 ALS

ALS is a neurodegenerative disease characterized by motor neuron degeneration and muscle atrophy. Studies suggest that α-actinin-2 expression is abnormal in ALS models, resulting in degenerative alterations at the neuromuscular junction. α-Actinin-2 binds to actin filaments through its ABD domain, forming antiparallel homodimers that help maintain the structure and function of the neuromuscular junction. In ALS models, abnormal α-actinin-2 expression leads to structural abnormalities at the neuromuscular junction, impairing motor neuron signaling and muscle contraction (Chen et al., 2024).

### 3.4 Other diseases

#### 3.4.1 Diabetes

Diabetes is a metabolic disease characterized by hyperglycemia. Studies indicate reduced α-actinin-2 expression in skeletal muscle of diabetic patients, impairing insulin sensitivity and glucose uptake (Hwang et al., 2010). α-Actinin-2 interacts with the insulin receptor through its EF-hand domain, thereby regulating insulin signaling pathways. In diabetic patients, decreased α-actinin-2 expression impairs insulin signaling, thereby impacting muscle insulin sensitivity and glucose uptake. Moreover, reduced α-actinin-2 may disrupt cytoskeletal stability and impair muscle cell metabolism (Hwang et al., 2010). These results provide novel insights into the pathogenesis of diabetes (Coletta and Mandarino, 2011; Aboalgasm et al., 2021a).

#### 3.4.2 Obesity

Obesity is a metabolic disease characterized by excessive weight gain. Studies demonstrate significantly reduced α-actinin-2 expression in skeletal muscle of obese individuals, impairing muscle metabolic function (Riedl et al., 2015). α-Actinin-2 regulates muscle metabolism by binding actin filaments through its ABD (Wolfarth et al., 2014). In patients with obesity, reduced expression of α-actinin-2 leads to abnormal muscle metabolic function, subsequently affecting energy expenditure and weight control (Hwang et al., 2010). These findings reveal the potential role of α-actinin-2 in obesity pathogenesis and offer a theoretical foundation for developing new therapeutic strategies (Coletta and Mandarino, 2011).

#### 3.4.3 Certain cancers

Abnormal expression levels of α-actinin-2 in certain cancers influence tumor invasion and metastasis. Studies indicate that α-actinin-2 is aberrantly expressed in breast cancer, thyroid cancer, colorectal cancer, and prostate cancer, where it affects tumor cell invasion and metastatic potential (Baris et al., 2005; Oikonomou et al., 2011). α-Actinin-2 regulates tumor cell motility by binding to actin filaments through its ABD domain. In some cancers, abnormal α-actinin-2 expression improves tumor cell motility, thereby promoting tumor invasion and metastasis (Weng et al., 2022). Data also indicate that overexpression of residues 383–632 in *ACTN2* inhibits tumor cell motility and proliferation by interfering with the interaction between angiogenin and α-actinin-2, indicating a potential mechanism for angiogenin’s role in tumor growth and metastasis (Hu et al., 2005).

#### 3.4.4 *ACTN2*-related myopathy

Recently, the role of new variants of α-actinin-2 in distal myopathies (actinomyelopathy subtype) has garnered increasing attention. However, three primary research gaps remain: First, the clinical significance of these new α-actinin-2 variants remains unclear. Second, a clear genotype-phenotype correlation for α-actinin-2-associated actinomyelopathy is lacking. Third, the pathological mechanisms highlighting these disorders have not been fully elucidated. To address these gaps, Johanna Ranta-Aho et al. employed a C2C12 cell model to functionally characterize multiple α-actinin-2 variants (frameshift and missense variants associated with dominant actinopathy) and to conduct association analyses with clinical data from patients harboring these variants. Results revealed that α-actinin-2 missense variants associated with recessive actinopathy did not induce detectable α-actinin-2 aggregates in the cellular model. Conversely, dominant-negative α-actinin-2 frameshift variants that cause protein elongation trigger abnormal intracellular aggregation of α-actinin-2. This suggests that such aggregation may represent a key pathological mechanism in some cases of dominant-negative actinopathy (Ranta-Aho et al., 2024b).

## 4 α-actinin-2 and disease diagnosis

Given the extensive involvement of α-actinin-2 in the pathogenesis of the aforementioned disorders, identifying specific genetic variants has become a critical component of precision medicine. Molecular diagnostics now complement traditional clinical assessments to refine disease classification and prognosis.

### 4.1 Skeletal muscle diseases

Mutations in the α-actinin-2 gene are associated with various skeletal muscle disorders, including distal myopathies, congenital myopathies, and muscular dystrophies (North and Beggs, 1996; Xia et al., 1997). These conditions typically manifest as muscle weakness, atrophy, and movement disorders. Muscle biopsy and genetic testing can identify specific α-actinin-2 mutations associated with distinct muscular disease phenotypes. For instance, the *p.Phe835Serfs66* mutation is correlated with distal myopathy, the *p.Ile134Asn* mutation with congenital myopathy, and the *p.Cys487Arg* mutation with muscular dystrophy. These results offer crucial molecular evidence for the early diagnosis of muscular disorders (Chen et al., 2021; Ranta-Aho et al., 2024a; Iruzubieta et al., 2025).

### 4.2 Cardiomyopathy

Cardiomyopathy is a group of cardiac diseases characterized by structural and functional abnormalities of the myocardium, primarily including HCM, DCM, and ARVC. Recently, mutations in the α-actinin-2 gene have become important molecular markers for diagnosing cardiomyopathy. Genetic sequencing technology can identify specific mutations associated with cardiomyopathy, such as *p.Met228Thr* and *p.Thr247Met*. These mutations typically occur within the ABD or spectrin-like repeat sequences, leading to defects in sarcomere assembly and cardiomyocyte hypertrophy (Atang et al., 2023; Noureddine et al., 2025). These comprehensive diagnostic approaches provide robust support for early intervention and management of cardiomyopathy.

## 5 α-actinin-2 as a therapeutic target

Currently, extensive clinical studies suggest that α-actinin-2 is deeply involved in the pathogenesis and progression of multiple disorders. Its crucial role in disease mechanisms opens novel therapeutic avenues and offers highly promising targets for associated disorders (**Table 3**).

**Table 3:** Potential therapeutic targets and strategies.

Disease	Mutation/context	Therapeutic target	Therapeutic strategy	Validation level	Ref.
HCM	*p.Met228Thr*	L-type Ca2+ channels	Channel Blockade (e.g., Diltiazem): Normalizes contractility and electrophysiological properties (AP duration).	Rat models; iPSC-CMs; Clinical Case Reports	(Norman et al., 2009; Haywood et al., 2016; Donkervoort et al., 2024)
DCM	*p.Glu583Ala*	Mitochondrial function	Metabolic Modulation (e.g., CoQ10): Enhances mitochondrial respiration and energy metabolism.	Knockout Rat; Cell culture	(Fan et al., 2018; Good et al., 2020)
ARVC	*p.Arg506Gly*	Calcium handling	Ion Regulation (e.g., Amiodarone): Restores intracellular calcium balance to suppress arrhythmias.	Knockout Rat; Cell culture	(Hou and Cortez, 2022; Xie et al., 2024)
Distal Myopathy	p.Phe835Serfs*66	Sarcomere stability	Sarcostability Enhancement: Stabilizes Z-disk structure to prevent contractile dysfunction.	Knockout Rat; Cell culture	(Lornage et al., 2019; Iruzubieta et al., 2025)
Congenital Myopathy	p.Ile134Asn	Actin binding	Binding Affinity Enhancement: Restores alpha-actinin-2 binding to actin filaments.	Knockout Rat; Cell culture	(Vainzof et al., 2002; Palop et al., 2005)
Muscular Dystrophy	*p.Cys487Arg*	Sarcolemma/Ca2+	Membrane Stabilization: Mitigates calcium-induced sarcolemmal instability.	Knockout Rat; Cell culture	(Vainzof et al., 2002; Hiroi et al., 2008)
Alzheimer’s (AD)	Reduced Expression	NMDA Receptor	Receptor Modulation (e.g., Memantine): Rescues synaptic plasticity and cognitive function.	Rat models; Cell culture	(Ratzliff and Soltesz, 2001)
ALS	Abnormal Expression	NMJ	Neuroprotection: Preserves NMJ integrity and delays motor neuron degeneration.	Rat models; Cell culture	(Coletta and Mandarino, 2011)
Diabetes/Obesity	Reduced Expression	Insulin Signaling	Insulin Sensitization (e.g., Metformin): Improves glucose uptake and muscle energy expenditure.	Rat models; Cell culture	(Bauce et al., 2000; Baris et al., 2005; Riedl et al., 2015)
Cancer Metastasis	Overexpression	Cytoskeleton/Motility	Motility Inhibition: Targets microtubule dynamics or NF-kappaB loop to suppress invasion.	Rat models; Cell culture	(Oikonomou et al., 2011; Ranta-Aho et al., 2024b)

### 5.1 Insulin resistance and diabetes

Insulin resistance in skeletal muscle is a hallmark of obesity and type 2 diabetes, involving the dysregulation of multiple physiological processes (Coletta and Mandarino, 2011). Recent studies suggest that inflammation, extracellular matrix remodeling, and changes in cytoskeletal proteins—including α-actinin-2—are closely correlated with insulin resistance. As a mechanical signal sensor, α-actinin-2 may contribute to the development of insulin resistance by regulating mitochondrial function and lipid metabolism. These findings provide novel insights into the pathological mechanisms of insulin resistance and offer potential therapeutic targets for associated diseases (Hwang et al., 2010).

Furthermore, hyperglycemia—a crucial metabolic abnormality in diabetes—is correlated with pathological cardiogenesis during embryonic development. However, its mechanisms of action and potential therapeutic targets remain elusive. Recent results are consistent with mechanisms involving abnormal Ca^2+^ handling and mitochondrial-dependent apoptosis, which are considered potential therapeutic targets for developmental diabetic cardiomyopathy (Aboalgasm et al., 2021b).

### 5.2 Interaction with angiogenin

Angiogenin is a factor involved in tumor angiogenesis. Studies demonstrate that α-actinin-2 directly interacts with Angiogenin, potentially contributing to tumor angiogenesis by regulating cell proliferation, movement, and invasion. This indicates that disrupting the α-actinin-2/Angiogenin interaction may inhibit tumor growth and metastasis, making it a potential novel anti-tumor target (Hu et al., 2005; Weng et al., 2022).

### 5.3 Gastric cancer metastasis

Bone marrow metastasis (BMM) in GC represents a highly invasive subtype with an extremely poor prognosis. Studies reveal that α-actinin-2 is specifically upregulated in GC, where the encoded α-actinin-2 improves metastatic capacity by promoting filopodia formation and actin filament cross-linking. Furthermore, α-actinin-2 forms a positive feedback regulatory loop with the NF-κB subunit RelA, further promoting its expression. These results demonstrate the crucial role of α-actinin-2 in GC BMM, indicating its potential as a therapeutic target and providing novel directions for GC treatment (Pinotsis et al., 2020; Wang et al., 2023).

### 5.4 HCM

HCM is a hereditary heart disease characterized by structural and contractile changes in the myocardium. In a patient with HCM, researchers identified a rare genetic mutation c.740C>T (p.T247M) located in the *ACTN2*, which encodes α-actinin-2. Using cardiomyocytes differentiated from patient-derived human induced pluripotent stem cells (hiPSCs) and engineered cardiac tissue, the study successfully simulated core pathological features of HCM, including cardiomyocyte hypertrophy, myofibrillar disarray, excessive contraction, impaired relaxation, high Ca^2+^ sensitivity of myofilaments, prolonged action potentials, and enhanced L-type Ca^2+^ currents (Prondzynski et al., 2019). Compared with controls, the L-type Ca^2+^ channel blocker diltiazem significantly decreased myocardial contractility, enhanced relaxation, and shortened action potential duration. Regarding these results, diltiazem therapy successfully enhanced QTc prolongation in affected family members. These findings not only offer compelling evidence for the pathogenic mechanism of α-actinin-2 gene mutations but also evaluate the immense potential of hiPSC technology for personalized therapy in cardiomyopathy (Atang et al., 2023).

## 6 Conclusion and future perspectives

α-Actinin-2 is a critical actin-binding protein that functions primarily as an antiparallel homodimer. Its distinct structural domains—the N-terminal ABD, central spectrin-like repeats, and C-terminal EF-hands—enable it to cross-link actin filaments at the sarcomeric Z-disk and interact with diverse signaling molecules, including NMDA receptors and CaMKII. As reviewed here, genetic variants in the *ACTN2* gene are strongly associated with the pathogenesis of hypertrophic and dilated cardiomyopathies, as well as specific skeletal myopathies and neurodegenerative disorders.

A synthesis of current findings reveals a divergence in pathogenic mechanisms based on tissue context. In striated muscle, disease phenotypes (e.g., HCM, DCM) typically arise from structural deficits: missense mutations (e.g., *p.Met228Thr*) impair the static cross-linking function of the Z-disk, leading to mechanical instability and contractile failure. Conversely, in non-muscle contexts such as gastric cancer metastasis, pathogenicity is driven by signaling dysregulation. Here, the overexpression of wild-type α-actinin-2 hijacks cell motility pathways (e.g., via the NF-κB feedback loop) to promote dynamic cytoskeletal remodeling. This comparison highlights that α-actinin-2 operates as a rigid stabilizer in muscle homeostasis but as a dynamic modulator in pathological cell migration.

Despite the identification of numerous pathogenic mutations, the precise molecular mechanisms linking specific structural alterations to clinical phenotypes remain to be fully elucidated. For instance, while diltiazem has shown promise in treating *ACTN2*-related HCM, effective targeted therapies for other *ACTN2*-associated conditions are currently lacking. However, the development of therapeutic interventions requires caution. Given the essential physiological role of wild-type α-actinin-2 in maintaining sarcomeric stability, broad inhibition or knockdown strategies could induce severe cardiotoxicity or myopathy. Therefore, therapeutic approaches must achieve high selectivity, targeting only pathogenic mutants or specific downstream signaling nodes without compromising basal protein function. Future pharmacological strategies could involve developing small-molecule allosteric modulators that specifically bind to the EF-hand domains. Such agents could precisely rectify the abnormal calcium sensitivity caused by mutations (e.g., p.Arg506Gly) without disrupting the protein’s core structural anchoring function at the Z-disk. Future research should focus on: (1) resolving the high-resolution structures of mutant α-actinin-2 dimers using cryo-electron microscopy to understand conformational changes; (2) investigating cell-type-specific regulatory mechanisms, particularly in non-muscle tissues, using single-cell transcriptomics; and (3) developing precision therapeutics that can specifically stabilize or correct the function of mutant α-actinin-2 proteins.

In conclusion, α-actinin-2 is essential for maintaining cytoskeletal integrity and cellular signaling. A more detailed understanding of its structure-function relationship will be vital for translating genetic findings into effective diagnostic and therapeutic strategies.

## Supplementary material

Supplementary Figure S1 Annual publication trend of α-actinin-2-related research (1993–2025). Data were retrieved from the PubMed database. The line graph illustrates the number of publications per year, highlighting a significant increase in research interest regarding disease associations and functional mechanisms over the last decade.
