# Supplementary material for: Biological properties of α-actinin-2 and its role and mechanisms in disease development
Source: Front Physiol. 2026 Apr 23;17:1794324. doi: 10.3389/fphys.2026.1794324 (PMC13149166; doi:10.3389/fphys.2026.1794324)
Supplement: Supplementary Figure S1 — Annual publication trend of α-actinin-2-related research (1993–2025). Data were retrieved from the PubMed database. The line graph illustrates the number of publications per year, highlighting a significant increase in research interest regarding disease associations and functional mechanisms over the last decade. [file Supplementaryfile1.docx]

Supplementary material

Fig S1. Displays the trend in the number of α-actinin-2-related studies in the PubMed database from 1993 to 2025, providing data support for our in-depth analysis of this trend.

Fig S1. PubMed Annual Publication Volume and Trend Chart, 1990–2025
